# Commoning the governance: a review of literature and the integration of power

**DOI:** 10.1007/s11625-022-01191-2

**Published:** 2022-08-15

**Authors:** Stefan Partelow, Aisa O. Manlosa

**Affiliations:** 1 Social Sciences Department, Leibniz Centre for Tropical Marine Research (ZMT), Fahrenheitstrasse 8, 28359 Bremen, Germany; 2 Faculty of Governance and Global Affairs, Leiden University, The Hague, Netherlands

**Keywords:** Institutional analysis, Institutions, Sustainability, Marine social science, Environmental governance

## Abstract

The concept of commoning is continuing to gain scholarly interest, with multiple definitions and interpretations across different research communities. In this article, we define commoning as the actions by groups with shared interests towards creating shared social and relational processes as the basis of governance strategy. Perhaps it can be more simply defined as collective ways of relating and governing. This article addresses two specific gaps in the commoning literature: (1) to bridge disparate strands of literature on commoning by briefly reviewing each and arguing for integration through epistemic pluralism, and (2) to explicitly examine how power is manifest in commoning processes by bringing in a framework on power (i.e., power over, power with, power to, power within) to understand the links between power and commoning governance processes in two case studies. The two cases are tourism governance on Gili Trawangan, Indonesia and aquatic food production systems in Bulacan, Philippines. We preface this analysis with the argument that power is an integral part of the commoning concept, but that it has yet to be analytically integrated to applications of the broader institutional analysis and development framework or within the networks of action situations approach. We argue that by making explicit how an analysis of power can be coupled to a network of action situations analysis in a qualitative way, we are advancing a key feature of the commoning concept, which we introduce as rooted in epistemic and analytical pluralism in the analysis of governance. In the discussion, we expand on how each case study reveals each of the four power dynamics, and how they improve the understanding of commoning as a pluralistic and perhaps bridging analytical concept.

## Introduction

This article reviews and applies the concept of *commoning* (a verb and social process), in contrast to commons (a substantive noun), as a useful tool to bridge different epistemic and analysis approaches in commons scholarship. However, within the literature on commoning, there are at least two differing strands. One strand refers to the processes of governance (i.e., institutional development and change) that lead to changing property rights for substantive commons. Substantive commons scholarship has primarily been focussed on whether common property rights (CPR) regimes and their associated governance mechanisms and institutions (e.g., rules, norms, rights) perform better than other rights regimes (e.g., private, state, open-access) in fostering sustainability outcomes in contexts with different social and ecological conditions (Ostrom 1990; Schlager and Ostrom 1992; Agrawal 2001). This is linked more to the notions of commonization and decommonization (Nayak 2021), referring to changes in CPR and public goods being governed under different property rights regimes. However, a critique of the substantive commons literature is that it often neglects important social and relational processes such as power, politicization and normative orientation towards sustainability (Bollier and Helfrich 2015; Cleaver and De Koning 2015; Clement et al. 2019; Nightingale 2019; Partelow et al. 2019). The other strand of commoning literature addresses these aspects, referring to commoning as the creation of a social commons itself, the emergence of collective social processes, distinct from the physical commons being governed, and necessary for creating the social conditions within which resource governance and collective action can occur. This is the understanding applied in this article. Thus, we define commoning as the actions by groups with shared interests towards creating shared social and relational processes as the basis of governance strategy, or more simply as collective ways of relating and governing (Nightingale 2019). Furthermore, we argue, that central to commoning are power dynamics, including the subjectivities and inter-personal relationships that interplay to create the complex social contexts within which governance mechanisms develop and can be changed.

This study aims to analyse networks of action situations using the concepts of commoning and power as complementary analytical lenses. In doing so, the article contributes to two gaps in the commoning literature: (1) to bridge disparate strands of literature on commoning by briefly reviewing each and arguing for integration through epistemic pluralism, and (2) to explicitly examine how power is manifest in commoning processes by bringing in a framework on power to understand the links between power and commons governance from a networks of action situation perspective in two case studies. We begin with introducing the networks of action situations and institutional analysis and development framework, and then review the diverse literature on commoning and explain our perspective on it. This is followed by a brief discussion on how we explicitly integrate power (i.e., coercive *power over*, collaborative *power with*, agentic *power to*, and psychic *power within*) (Rowlands 1998; Chambers 2006) in analysing commoning processes. These perspectives are then applied to two empirical case studies of coastal commons governance in Indonesia and the Philippines.

### Networks of action situations

**Fig. 1:**
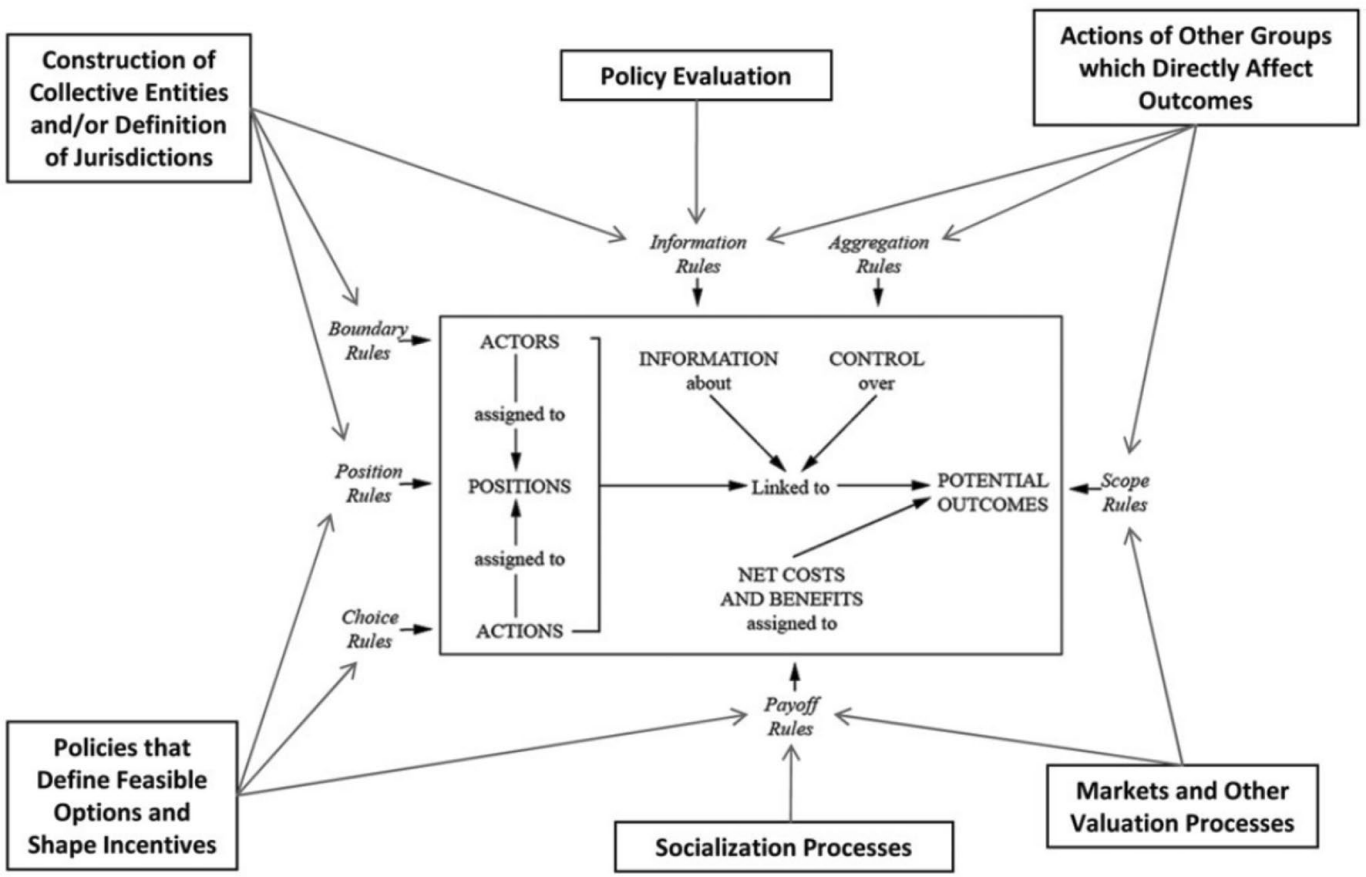
An action situation (center) within the institutional analysis and development (IAD) framework, and its influences from a network of adjacent action situations (outer boxes). Figure taken directly from McGinnis (2011a, p. 54)

Action situations in environmental governance are referred to as “social spaces where individuals interact, exchange goods and services, solve problems, dominate one another, or fight,” (Ostrom 2011, p. 11), in their efforts to use, provision, and govern shared resources. The concept allows an analyst “to isolate the immediate structure affecting a process of interest […] for the purpose of explaining regularities in human actions and results, and potentially to reform them,” (Ostrom 2011, p. 11). These include the rules and positions different actors have as well as the information about, control over, and costs and benefits for each actor in the action situation (McGinnis 2011a). The network approach also extends analysis to the interactions among multiple action situations in a governance system. McGinnis (2011a, b) argues that “the working components of an action situation can be usefully interpreted as the outcomes of processes occurring in adjacent action situations,” and that action situations are adjacent if an outcome in one influences any variable in the others. Analysing linkages between multiple action situations is thus useful because it enables an analyst to explore co-dependencies across different institutions with a detailed framework (Fig. 1), which has already been demonstrated in a rich body of literature (McGinnis 2011a; Ostrom 2011; Kimmich 2013; Lubell 2013; Villamayor-Tomas et al. 2015; Oberlack et al. 2018; Berardo and Lubell 2019; Möck et al. 2019). A comprehensive and recent review of the literature has been undertaken by Kimmich et al. (2022) for this Special Issue.

While action situation analyses have focussed on identifying, isolating and describing the constituent pieces influencing actor behavior (e.g., their positions, rules, motivations), essential for institutional analysis (Table 1), they are less able to analyse the role of subjectivity, social or relational processes in the spaces between those constituent parts, and how they change over time. Understanding the collective nature of governing is often missing, including subjective experiences and power dynamics. In other words, achieving social and ecologically successful commons outcomes is not only influenced by the exogenous variables and internal dynamics of individual action situations, or their networks, but also the experiences of individuals, their relationships with each other and their intentions. Such relationship processes create power dynamics that alter how people perceive and behave within action situations. How rules and positions influence actors cannot only be taken at face value as actors’ perceptions of them and their relationships with others involved play important roles in how a rule or position influences behavior of any individual, a group, and the outcome.

**Table 1:** Rules that specify the values of the working components of an action situation; each rule has emerged as the outcome of interactions in an adjacent action situation at a different level of analysis or arena of choice (McGinnis 2011b)

Position/choice rules	Specify a set of positions, each of which has a unique combination of resources, opportunities, preferences, and responsibilities
Boundary rules	Specify how participants enter or leave these positions
Authority rules	Specify which set of actions is assigned to which position
Aggregation rules	Specify the transformation function from actions to intermediate or final outcomes
Scope rules	Specify a set of outcomes
Information rules	Specify the information available to each position
Payoff rules	Specify how benefits and costs are required, permitted, or forbidden to players

These dimensions can, in part, be better understood and analysed by combining analytical traditions and epistemic pluralism embodied in commoning. Below, we review the commoning literature and its pluralism, and introduce a framework of power for unpacking commoning dynamics to position our empirical analysis of two case studies.

### Commoning: a review of literature and perspectives

One challenge with commoning as a concept is that it attempts to bridge epistemological and ontological differences between numerous fields, disciplines and schools of thought in the commons literature such as sociology, institutional economics, political science and human geography. Rather than seeing these as incompatible, our view of commoning seeks integration through epistemic pluralism such as in critical realism (Bhaskar 2013). To exemplify this, let’s simplify and say that there are three main philosophies of science: positivism, interpretivism and social constructivism. The latter two (i.e., the sociology-oriented perspectives) adopt a form of epistemic relativism that we are all so intertwined in our own particular set of stories, relationships (to people and objects) and language games that there is no basis for distinguishing between the objects of science and the practice of science, as they are created social realities (Gorski 2013). In contrast, positivism in its basic form draws no ontological distinction between natural and social realms, both are neutrally observable and therefore any claims about them are falsifiable, including experience, regardless of subjectivity (Gorski 2013). Nowadays, few would likely defend either as fully true, and many see the logic and value of each, but struggle to reconcile or integrate them. We observe this in environmental governance scholarship generally, that more recently evolved theories are combinatory (Partelow et al. 2020). However, while combinatory or integrated philosophies of science do exist, they are more slowly adopted with more challenges. Most notably, critical realism (Collier 1994; Bhaskar 2013), in our view, is a philosophy that may help position commoning as an analytical concept to create integrative bridges across scholarship. Critical realism reconciles numerous issues such as the natural and social understanding of causality, and relationships between structures and agents in systems as not being constant or entirely generalizable, but also not fully derived from actor intentions. Below we review the different commoning literatures, with interest to seek integration and pluralism.

Commoning builds on and compliments the study of commons governance with relational sociology (Emirbayer 1997) and critical institutionalism (Cleaver and De Koning 2015). Cleaver and Koning (2015) suggest critical institutionalism as, drawing from critical realism (Bhaskar 2013), exploring “how institutions dynamically mediate relationships between people, natural resources and society, […] the power relations that animate them; [while] a social justice lens is often used to scrutinize the outcomes of institutional processes,” (p. 1). In similar fashion, Emirbayer (1997) expresses a tension in sociology, that “sociologists today are faced with a fundamental dilemma: whether to conceive of the social world as consisting primarily in substances or in processes, in static ‘things’ or in dynamic, unfolding relations,” (p. 281).

In another strand of commons scholarship, Nayak and Berkes (2011) suggest commonization and decommonization as “a process through which a resource gets converted into a jointly used resource [or] a process through which a jointly used resource under commons institutions loses these essential characteristics,” (p. 133). Here, any resource can enter a process of commonization, and in their case study of fisheries in Chilika Lagoon, India they outline how processes of (de-)commonization relate to issues of social justice. In an edited volume (Nayak 2021), commonization and decommonization are explored in numerous case studies to understand commons as a process of constant and evolving institutional change, shifting how and why resources are used, provisioned and governed under common property regimes over time. In one of the book’s chapters, Basurto and Lozano (2021) refer to commoning as “an explicitly relational concept that has emerged in more critical engagements with the commons to emphasize process and embodied forms of praxis as central for the maintenance and formation of commons,” and that the concept represents “the constant coming together of humans […] a constantly changing and evolving relationality between humans, non-humans, their territories and histories, and the forging of subjectivities that ultimately give meaning to issues.”

There are recurrent themes underlying the ways in which commoning has been described by different scholars. These include the centrality of relationships in social processes, the dynamism of such relationships, and the subjectivities that are inherent in these relationships. There are also commons as an entity versus commoning as an action of creating social commons and the social organization that governs them.

More simply, Euler (2018) states that “commoning creates commons” and that “the social practices of commoning are argued to be voluntary and inclusively self-organized activities and mediation of peers who aim at satisfying needs,” (p. 10). Furthermore, Bollier (2020) refers to commoning as a notion that “cultivates new cultural spaces and nourishes inner, subjective experiences that have far more to do with the human condition and social change,” (p. 4) and that it is “a dynamic, evolving social activity” (p. 6). Numerous scholars refer to Linebaugh’s (2008) reflection that “to speak of the commons as if it were a natural resource is misleading at best and dangerous at worst—the commons is an activity and, if anything, it expresses relationships in society that are inseparable from relations to nature. It might be better to keep the word as a verb, an activity, rather than as a noun, a substantive,” (p. 279) (Gibson-Graham et al. 2016; Blaser and De La Cadena 2017). Blaser and De La Cadena (2017) also draw on Bollier’s work noting that “commons is a process of creating and nurturing community,” (p. 186). In outlining their notion of the ‘uncommons’, they prime readers with questions of how far shared domains of communities extend, raising questions of scale and inclusion. They also ask: what do the commons and uncommons include, and with what responsibilities?

Fournier (2013) proposes a threefold view of commoning as societal organization either *in*, *of* or *for* the common. She further notes a “creative potential of commoning, the fact that the commons (as patterns of social relations unmediated by the market) are produced through the process of using things in common,” (p. 448). Federici (2011) draws on feminist perspectives to position commoning similarly in relation to production, stating that “if commoning has any meaning, it must be the production of ourselves as a common subject. This is how we must understand the slogan ‘no commons without community.’ […] as a quality of relations, a principle of cooperation and of responsibility to each other and to the earth,” (p. 7). Both authors draw on production, not to produce substantive commons, but relational communities.

From human geography and political ecology, which numerous of the above authors intersect with, commoning has drawn substantial interest in thinking about place, sharedness and community. Nikolaeva et al. (2019) reflect that “within geography, the notion of commoning is primarily engaged through two debates: the discussion of the management of CPR beyond the state and the market, and the interrogation of the notions of commons and commoning as tools to envision and enact alternative post‐capitalist politics,” (p. 352). More specifically, Nightingale (2019) centers power as a key part of commoning, arguing that societal transformation should aim for “doing commoning, becoming in common, rather than seeking to cement property rights, [thus enabling] relations of sharing and collective practices as the backbone of durable commoning efforts,” (p. 16). Singh (2017) notes that “a growing number of commons activists suggest that diverse commoning projects represent an alternative form of production in the making and are reminders that alternative social relations are entirely thinkable” (p. 753). Later elaborating that there is a need for “becoming a commoner…that the commons can be conceptualized as a site of affective socio-nature encounters…that can foster subjectivities of ‘being in common’ with others” (p. 754). Further work has emphasized how commoning can create shared spaces and knowledge production, often in relation to re-thinking political and economic models that enable empowering communities of commoners as a shift beyond narratives and practices of the individual-state political economy.

In this article, we view commoning as the actions by groups with shared interests towards creating shared social and relational processes as the basis of governance strategy. What is of interest are the emergence and maintenance of collective social processes of governing, and the perceptions and experiences of participants doing the commoning. For example, is their relationship to governing processes, and to the others involved, an experience of empowerment and agency, or rather of marginalization and disempowerment? Power dynamics are important for understanding such processes, and power dynamics more broadly are increasingly recognized as important in environmental governance (Boonstra 2016; Van Assche et al. 2017; Morrison et al. 2019), but lack integration into commons and institutional analysis literature (Epstein et al. 2014; Cornea et al. 2016; Cleaver and Whaley 2018; Mudliar and Koontz 2021).

### Commoning and power

Power is a central feature of social systems, yet its integration as an analytical concept within commons scholarship remains largely absent in the field’s prominent frameworks (Cox et al. 2010, 2021; McGinnis 2011b; Partelow 2018). Power is a social phenomenon that animates social relations and is inevitably inscribed in governance processes through institutions (Levi 1990; Lukes 2004). The institutions or rules that shape governance dynamics determine who gets to participate in decision-making processes, to what extent, how, and which matters are decided and acted on (Ostrom 2011). These processes, in turn, determine terms of access and control over resources, and how benefits are distributed (Kabeer 1999).

Power is understood in different ways (Table 2), but the mainstream understanding is that of *power over,* or the capacity to influence, coerce, or force others contrary to the latter’s will overtly or covertly (VeneKlasen and Miller 2007). Distinctions between the powerful and powerless are based on who has more (or less) *power over* (Rowlands 1998; Chambers 2006; Gaventa 2006). This form of power functions as a zero-sum game in which the increase of power by some leads to a reduction of power in others. In this sense, power is not neutral but involves a distributional aspect (Rowlands 1995). However, power has also been investigated beyond power over (Rowlands 1998; Chambers 2006). It has been conceptualized in terms of *power with,* in which individuals come together as a group (e.g., coalitions, alliances) and amplify their voices to effect change. Another understanding of power is in terms of both individual and collective agency. *Power to*, is the generation of abilities by individuals or groups to choose, act, and realize desired outcomes. Finally, there is also *power within,* which emanates from individuals’ internal resources and involves people’s sense of dignity and self-efficacy.

**Table 2:** Basic analytical framework for power outlined in Chambers (2006) and VeneKlasen and Miller (2007)

*Coercive* power over	The capacity of actors to exert control or influence over others overtly or implicitly
*Collaborative* power with	Coalitions or alliances where individuals come together to effect change
*Agentic* power to	The generation of abilities to choose and realize desired outcomes
*Psychic* power within	Individuals’ internal resources and involves people’s sense of dignity and self-efficacy

Power asymmetries manifesting as *power over* in which few actors are able to exert strong influence over decision-making processes and distribution of benefits are typically associated with hierarchical governance structures and exclusionary governance processes that privilege the participation and control of the elite. However, polycentric governance arrangements and hybrid forms also exist which are conducive to strengthening *power with* amongst collaborating actors by foregrounding participation (e.g., Acton and Gruby 2021). Power as control tends to be viewed as undesirable because of its tendency to lead to marginalization of less powerful actors (e.g., Borras et al. 2011). However, Chambers (2006) sees the different forms of power and their interactions as opportunities. He views these inevitable interactions as ones that can be facilitated to bring about desired results. For instance, the *power over* held by government actors and maintained through formal institutions can be harnessed to create favourable conditions for collaboration and empowerment in communities as is the case in hybrid governance arrangements (e.g., Koppenjan et al. 2019).

## Methods

This research takes a case study approach, selecting two coastal social-ecological systems in tropical countries where the authors have conducted empirical work (i.e., Gili Trawangan, Indonesia and Bulacan, Philippines). The main livelihoods in these two systems differ, with ecotourism in Gili Trawangan, Indonesia and aquatic food production from both capture fisheries and aquaculture in Bulacan, Philippines. In both cases, the networks of action situations are described and analysed with an analytical framework of power in processes of commoning (or de-commoning).

### Ecotourism in Gili Trawangan, Indonesia

Data were collected during multiple field work phases in April 2017, November 2018 and October 2020. More than 100 semi-structured interviews were conducted (SP). All interviews were conducted in English, transcribed and analysed for their respective projects (Partelow and Nelson 2018; Partelow 2020). Questions guiding the research include institutional analysis, actor networks, community disaster resilience and the evolution of self-organized governance through collective action. The data used in this article draws on insights across the whole time period. A review of all known literature on the Gili Islands is provided, based on a systematic search on SCOPUS, Web of Science Core Collection and Google Scholar. This reviewed literature has also guided the analysis, as well as an on-going transdisciplinary partnership with a local NGO the Gili EcoTrust (http://giliecotrust.com/), the Indonesia Biru Foundation (https://indonesia-biru.com/) and a prior partnership with the Gili Shark Foundation (https://www.gilisharkconservation.com/).

### Aquatic food systems in Bulacan, Philippines

The case study in the province of Bulacan, Philippines is based on an empirical study that examined coastal social-ecological changes where aquaculture has become an important food sector interacting with capture fisheries. Qualitative primary data was collected in the period from November 2019 to March 2020 on institutional changes over time. Data collection included 67 in-depth interviews (AOM) with fishers, fish farmers, aquatic food production workers, market actors and state representatives, with assistance from local gatekeepers, government staff, and local aquatic food producers. Interviews were conducted in the Tagalog language, recorded, and transcribed into English. Data from interviews were further complemented by a thematic analysis of institutional documents (e.g., Municipal Fisheries Ordinances of the municipalities of Hagonoy, Paombong, and Malolos), Philippine Fisheries Code), and by participant observation.

### Data analysis

This study drew on qualitative data from the above field work and findings from previous published research. As a first step, we generated (i) qualitative descriptions of how resources were used and managed in each case. We included both formal and informal social processes shaping coastal research governance. We then identified (ii) networks of action situations of key importance. Within each action situation, we investigated the (iii) forms of power that manifested in social interactions, and as a final step (iv) how commoning unfolds.

## Findings

### Tourism, commoning and power in Gili Trawangan, Indonesia

Gili Trawangan is a ~ 4 km^2^ tourism island off the coast of Lombok, Indonesia. The economy is rooted in SCUBA diving accessible coral reefs, dating back to the early 1990s (Satria et al. 2006; Charlie et al. 2012; Graci 2013; Hampton and Jeyacheya 2015), and has flourished into an international beach and nightlife destination. Before the COVID-19 pandemic, the island had an estimated 750 businesses with 2,500 permanent residents (Partelow and Nelson 2018; Partelow 2020). The island has no motorized vehicles, only horse carts, and is only accessible by boat from Bali or Lombok. The island faces numerous governance problems that are centered around three main action situations: (1) self-organization, (2) coral reef use, (3) waste production and management (Table 3).

**Table 3:** Actors and action situations on Gili Trawangan, Indonesia

Actor types	Primary motivations	Action situation 1: self-organization	Action situation 2: coral reef use	Action situation 3: waste production and management
SCUBA diving businesses (+ 30)	Profitable and safe diving	Reef monitoring, safety rules for diving. Financially support NGOs. Develop standards and economic cooperation	Intensive use for profit	Produce waste with local repercussions. Self-organize management
Local NGOs (GET, GSC, FMPL, GIDA)	Conservation, coordination, operational tasks	Develop waste management and conservation programs. Information campaigns, raising money and political support. Organize actor interests	Management, restoration and monitoring	Manage waste, information and action programs. Coordinate actors
Tourists (up to 1 million per year)	Enjoyable experience, lowest price	Vacation spending, giving ratings and social media posts	Use for enjoyment	Produce waste without repercussions
Hotel, bars and restaurants (+ 700)	Profit	Cooperate with dive shops. Advertising internationally	Indirect use	Produce waste with local repercussions
Local Indonesian employees (up to 1000)	Sustainable livelihood	Support Lombok families and economy	Use via diving guide and fishing	Produce waste
Foreign owners and employees (200 +)	Sustainable livelihood	Main contributors of financing, social, economic and environmental impacts, but also problem solving	Use via diving guide	Produce waste
Local and regional government (Island head, elite families)	Social and political stability. Maintaining authority	Coordinates business activities, enforces economic rules. Coordinates with regional and national govt. Lobbies for island investments	Monitoring Gili MATra marine park. Re-zoning processes and rule formation	Operational oversight duties
National government	Policy making and enforcement	Develops economic and immigration rules. Direct enforcement or coordination with local govt. Tax collection and allocation. Economic strategy. Political oversight. Information sharing	Supports/recognizes formal marine park (Gili MATra)	Funds major infrastructure projects

The three action situations on Gili Trawangan are highly networked. Self-organization is an overarching social necessity on the island to facilitate economic development, to address social and environmental issues and to make life on the island enjoyable. Coral reef use and waste management are shaped by the island’s overall self-organizational history among a wide range of actors, however, each can be seen as its own action situation due to different configurations of actors, rules, motivations and outcomes.

#### Self-organizing activities

Self-organization is the cornerstone of the island’s economic development. Lacking government involvement, collective action activities are inclusive of many actors that influence coral reef use and waste management. Nearly all public infrastructure, social learning activities, economic and social services need to be self-organized by businesses, residents and heads of the island including schools, waste management, sewage, on-island transportation, environmental conservation, safety, medical services and social-political organization (Willmott and Graci 2012; Partelow and Nelson 2018; Nelson et al. 2019, 2021; Partelow 2020). Knowledge on other procedures such as permitting processes, hiring labour, construction strategies, supply chain and goods acquisitions are held as valuable local knowledge and collectively shared. Self-organizing activities as informal sharing has manifested into *power with* the EcoTrust, Gili Island Dive Association (GIDA), Front Masyarakat Peduli Lingkungan—Community Front for Environmental Care (FMPL), and other community associations.

The national government has funded larger infrastructure projects such as a waste processing facility, roads, local police, a pier and sewage systems. However, the local government (regency level) is tasked with day-to-day oversight, although involvement is minimal. Coordination between governments and island stakeholders to establish *power with*, is a continual challenge of trying to foster regular and transparent communication. Governments have finances and authority, and the *power to* make decisions, but are often disconnected from local realities and lack incentives to act quickly. On the other side, locals understand what needs to be done but often lack the financing or legal authority (*power to*). Local elite Indonesian families have historically had substantial *power over* island governance and island-government relations. Another challenge is that most of the larger, older and influential businesses are foreign-owned and managed, providing substantial tax revenue (Partelow and Nelson 2020). However, the island receives little locally useful public investment or knowledge of where taxed revenue is allocated (Partelow and Nelson 2018). Foreign owners would like to have more representation given their role in driving development, but have little influence. Meanwhile, the 200 local families (non-elites, many poor) living on the island, have little *power to* voice or influence island activities, and seem to be the most overlooked group despite their important small businesses (e.g., prepared food, crafts). Nonetheless, nearly all groups have developed a strong *power within,* to deal with joint challenges, cooperate and support low income locals, and many share a common vision for sustaining Gili Trawangan. Nonetheless all businesses have interests in profitability and securing investments, and given the island’s economic prosperity, it has drawn substantial outside investment into larger hotel chains disconnected from the *power within* community identity of collective activities and stewardship.

Local customs, formal associations and community groups largely shape rules and norms for governing. Commoning activities are essential for day-to-day governing, including building social capital, forming trust, building inter-personal relationships and building community networks among businesses, island associations and elite families (Kamsma and Bras 2000; Satria et al. 2006; Graci 2008, 2013; Bottema and Bush 2012; Charlie et al. 2012; Rianto 2014; Hampton and Jeyacheya 2015; Partelow and Nelson 2018; Partelow 2020). While governments have formal *power over* the island, the EcoTrust has the *power to* from the community to actualize daily activities in the form of *power with* business owners and influential elite families. Importantly, many individuals who have been on the island have developed a sense of *power within*, solving collective action problems and dealing with constant challenges.

There are two competing logics in these power dynamics. First, there is a nearly universal sense of sharedness and recognition of the need to work together with held power, and that creating and maintaining the sense of a shared social momentum is essential for sustaining the island and individual businesses as a result of them. These commoning activities are essential for using held power for supporting community goals, a key feature of the island’s informal social contract. However, constant challenges exist including self-interest, elite capture and foreign-local economic partnerships. For example, foreign owners have *power over* financing and operations, while Indonesian partners have *power over* legitimacy and legal issues.

The second competing logic is the maintenance of the power each group has. Power comes with influence and often economic opportunities, even in the case of EcoTrust in garnering funding and support. A critique here is that *power to* influence activities and the opportunity to take part in *power with*, is limited to a close inner circle of older business owners and managers who have strong trust and joint interests, and who informally discuss strategies and activities outside of the formal associations. This happens in part because they are the few who provide substantial financial backing and can make practical needs happen (e.g., procuring goods, local approvals through personal connections), and are thus major contributors to solving collective action challenges on the island on important environmental and social issues (Partelow 2020). They have also started the EcoTrust, funded a substantial amount of the island’s artificial reef installations, and have lobbied local government on numerous issues including boardwalk access restrictions. However, for new business owners, learning these norms and supporting the EcoTrust is expected, while also discovering and then accepting, who has power, which are the foreign mostly European and Australian owners and managers (although this is changing to a more diverse group of foreign owners and some fully Indonesian businesses). Trust needs to be built with every new business and person, which the island’s social landscape and culture seems to cultivate well and quickly, and in a mostly positive way. From the outside, the narrative is two-sided. On one hand, the inner circle has been the catalyst for collective action and progress, on the other hand, it creates an in-group dynamic that draws non-inclusive skepticism and raises transparency issues as the island grows.

#### Coral reef use

**Fig. 2:**
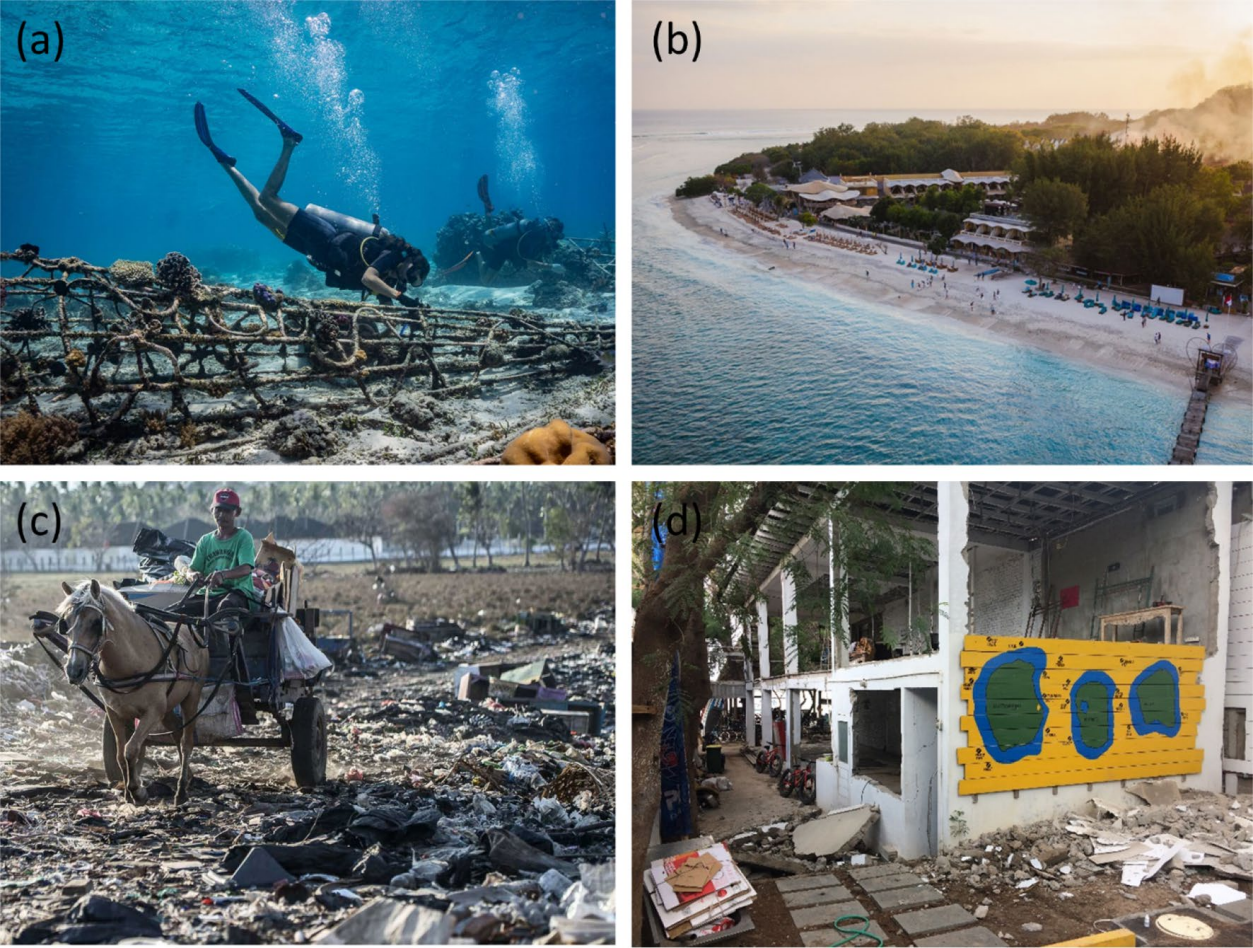
**a** SCUBA divers attach coral fragments onto an artificial reef structure, providing restoration habitat and erosion protection for the island’s coast. **b** Aerial view of beachfront resort on southeast Gili Trawangan. **c** Horse cart driver collecting waste and delivering it to the dump in the center of the island. **d** Destroyed SCUBA center from the August 2018 earthquakes. Photos **a**, **b** and **c** from the Gili EcoTrust, and photo **d** from authors

The Gili EcoTrust was founded in 2002 by the island’s main dive shops (mostly foreigners) at the time to protect nearby coral reefs from destructive fishing, eventually leading to the development of a zoned marine park (Gill MATra) around all three islands. The EcoTrust is funded through encouraging tourists to make a one-time donation (~ $4 USD) upon registration at each supporting dive business for recreational SCUBA diving. Donations directly support employees and other activities such as placing mooring buoys to limit anchoring, developing reef restoration programs and other sustainable tourism projects (Fig. 2).

The EcoTrust now has *power over* nearly all island environmental, governance and political activities, gaining influence by developing *power with* the island’s stakeholders. The EcoTrust has a social license to operate through building trust, community networks and social capital over time (Partelow 2020). Another self-organized industry association was created in 2010 due to rapid tourism and business growth, the Gili Island Dive Association (GIDA), to organize cooperation on water safety and economic issues. Coral reefs around the island now face crowding and overuse issues from more than 30 SCUBA businesses, snorkelers, anchoring and bleaching events (before COVID-19). GIDA was founded and supported by older SCUBA businesses and has gained *power over* water operations and industry economic cooperation (e.g., safety standardization, price agreements, rotational event nights, fees, marketing). The organization and its meetings have also become social events and spaces for discussing other issues.

#### Waste production and management

Waste production and management is the most recurrent issue, with numerous management efforts over years led by EcoTrust, local businesses, private initiatives and heads of the island. Nearly all have been fraught with challenges, balancing what is locally effective versus legal and formal in the puzzle of political relationships. Waste issues include leadership, trust and generating consensus on responsibilities, fees, efficiency, compliance and what to do with the waste. For example, local elite Indonesian families have the *power to* put in place the head of the island, often a person who would bear risk as the formal head of island while still being guided behind the scenes by elite families who also have *power over* relationships with local government, local people and business affairs. The national government has overarching authority, *power over* financing major infrastructure projects (e.g., collection and recycling center) and legal issues. Local government has the *power to* operationalize those projects, collect taxes, and enforce rules. However, this social license has given the EcoTrust local *power to* operate, and holds *power with* the island community. Waste management has evolved since, and is now run by a local Indonesian organization called FMPL, supported by the EcoTrust. However, the island’s central dump remains a critical issue. As of 2021, the national government has funded the construction of a waste processing facility, under consultation from EcoTrust, but only government officials can operate it, but none have been provided. Current FMPL programs continue to use the regular dump, threatening health around the island due to leakage and burning.

### Aquatic food production, commoning and power in Bulacan, Philippines

Bulacan is an important area for capture fisheries and aquaculture in the northern part of the Philippines some 40 km from the capital Metro Manila. The case study includes the municipalities of Hagonoy, Paombong and Malolos off the coast of Manila Bay.

**Fig. 3:**
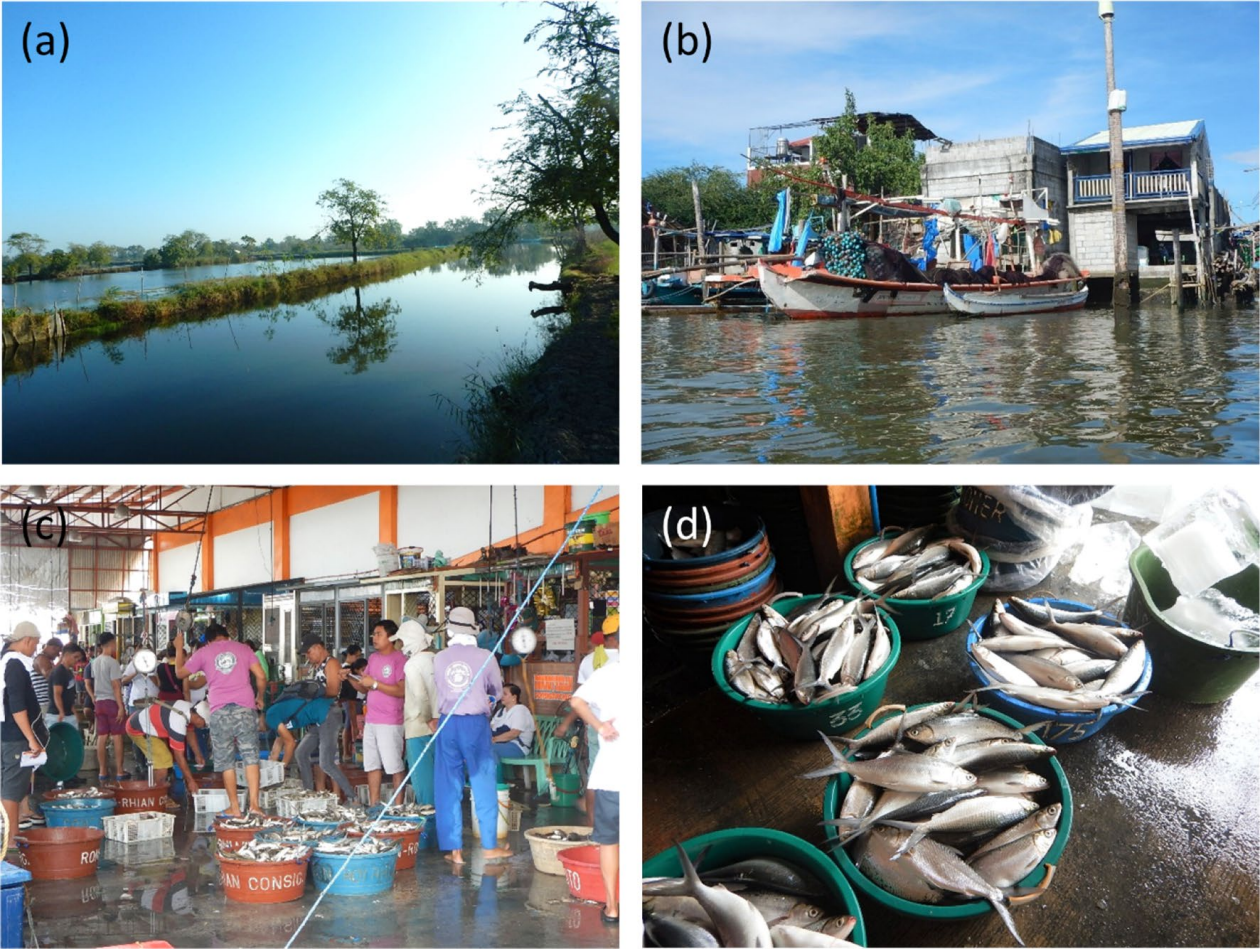
Sights from Bulacan, Philippines. **a** A small-scale brackish water fish pond in Paombong. **b** Homes and fishing boats in Hagonoy. **c** Public fish market in Hagonoy. **d** Milkfish being sold in a fish market in Malolos

Small scale fishing is undertaken in estuaries and municipal waters. Aquaculture is done in brackish water earthen ponds and spans non-intensive to intensive production along the coast and inland (Fig. 3). Fish ponds primarily produce milkfish, tilapia, shrimp, and mudcrabs. The sustainability challenges in this case study include environmental degradation (e.g., water pollution from intensive aquaculture), economic inequities from market arrangements, and the empowerment of small-scale aquatic food producers (Manlosa et al. 2021).

The network of action situations which influence sustainability in aquatic food production are (1) rule and norm-making for environmental management, (2) marketing of aquatic food, and (3) establishment of associations of food producers. Information on this network of action situations are summarized in Table 4, with associated direct quotes in Table 5.

**Table 4:** Description of actors and action situations in aquatic food production in Bulacan, Philippines

Actor types	Primary motivations	Action situation 1: rule and norm-making for environmental management	Action situation 2: marketing of aquatic food	Action situation 3: establishment of community associations by and for small scale aquatic food producers
Artisanal fishers	Sustainable livelihood, income to support family, food for family	Representation in the formulation of municipal fisheries ordinances, participation in enforcement of local regulations for capture fishers as Bantay Dagat (sea guards), compliance with various regulations including on the use of fishing gears, registration of stationary fish traps, and fishing in prescribed fishing grounds	Provision of caught fish and other aquatic food to local fish markets, arrangements with middlemen (locally called consignacions) (e.g. taking out loan)	Membership in fishers’ organizations, participation in meetings and discussions in fishers’ organizations, election of and collaboration with group leaders who can represent other fishers in both local and higher scale governance processes, participation in decision-making about the purpose and activities of the group, financial contribution to group funds
Smallholder fish farmers	Access capital and invest in fish farms to produce food and generate profit	Representation in the formulation of municipal fisheries ordinances, participation in state-sponsored capacity development activities for environmentally friendly aquaculture production, influence other fish farmers to stop using toxic chemicals during pond preparation	Provision of farmed fish and other aquatic food to local fish markets, arrangements with middlemen (locally called consignacions) (e.g. taking out loan)	Membership in fish farmers’ organizations, participation in meetings and discussions in fish farmers’ organizations, election of and collaboration with group leaders who can represent other fish famers in both local and higher scale governance processes, participation in decision-making about the purpose and activities of the group, financial contribution to group funds
Bureau of Fisheries and Aquatic Resources (BFAR) Regional Office	Higher level governance of aquatic resources (e.g. national and regional) to promote food security, poverty alleviation, and economic development through aquatic food production	Design and implement livelihood assistance projects and other development programs for artisanal fishers and small scale fish farmers, collaborate with other state and community actors to enforce Philippine Fisheries Code	Certification of large-scale aquaculture producers and large fish markets, investment in the construction of Community Fish Landing Centers (CFLC), provision of support for small scale aquatic food producers through capacity development activities in fish processing and value-adding and through access to cold storage facilities, issuance of local transport permits for aquatic food to be transported into other cities and towns	Provide administrative guidance, financial resources, and logistical support for regional meetings of leaders of fishers and fish farmers associations
Local Government Units	Manage aquatic resources for local economic development	Formulation and implementation of Municipal Fisheries Ordinances which contain locally contextualized rules for aquatic food production	Implementation of rules for business registration, issuance of business permits and local transport permits for aquatic food businesses, regular collection of registration fees from market actors particularly middlemen (consignacions) and local vendors	Facilitate formal registration of community-level fishers’ and fish farmers’ associations, work with community associations in the implementation of various projects
Middlemen/consignacions	Buy the highest volume and best quality of fish supply from fishers and fish farmers and sell at most profitable price	Formation of an association of consignacions, formulation of rules and by-laws for the operation of local fish markets	Purchase of fish from aquatic food producers, fish auctions, provision of credit to fishers and fish farmers when needed for livelihoods or personal needs, informal arrangements, cultural norms (e.g. suki or trusted buyer/seller relationships, utang or borrowed money), pay-later arrangements, patron-client relations	Not applicable

**Table 5:** Illustrative quotes on forms of power in action situations from the two case studies

Forms of power	Gili Trawangan, Indonesia	Bulacan, Philippines
Power over	“[NGOs and businesses are] giving [local government] transparency every month, we’re giving them a good example and pushing them, but there is pride and power there, they are the authorities, they have the power to enforce the law they have the power to decide to who those facilities will go to, so at the end of it we’re just suggesting things and waiting for them to react” (*Local NGO*) (Action situation 3) “for the government we are propose something that makes sense for the community or for business or for tourism, but then they don’t get it and then they don’t end up doing it, and we end up doing it, yeah it happens a lot” (*Indonesian business owner*) (Action situation 1)	“When large-scale intensive aquaculture flourished, smallholder aquatic food producers felt the effects and undertook mass action. Various aquatic species disappeared such as shellfishes. There were protests and a public hearing in Hagonoy. The mayor called the large fish pond owners. But they declared ‚This is private property’, you can’t stop them… how will you stop that?” (*Staff, Hagonoy Municipal Agriculture Office*) (*Action Situation 1*)
Power with	“there’s always been a handful of us, the main five or six shops that have been here the longest, because I really agree with them and we call it mini GIDA, basically if something comes up and you’ve been asked to deal with it, and you’re not really sure how to do it […] you could sit down with these six people instead, you know it’s not really mafioso style, but more to support you.” (*Foreign SCUBA business owner/GIDA member*) (Action situation 2)	“Our group is open in sharing ideas and responsibilities. Even with financial matters. When we no longer have money, we say ‘We have no money now. And we need this. What should we do?’ Then let’s bring out money. How much should we bring out? One can say ‘I can only afford to give this.’… If you are able to give more, good. If there is someone who has nothing to give, then it can be pulled by those who give more. Even if you are not bringing anything, you should join the meeting. Be with the group. That is what we did.” (*Fish farmer, Paombong*) (Action situation 3)
Power to	“since the earthquake, it reset a lot of relationships among, let’s call them the powerful locals, and us Westerners. And they saw that, you know, the support we gave them, and we’ll work together. And it brought us all back together […] building projects and stuff like that, but more financially to, you know, some of their family members working amongst the businesses, but also just someone to sit and talk to. And that works both ways. […] a sort of internal support.” (*Foreign SCUBA business manager*) (Action situation 1) “There are many organizations of local people, they try to meet and discuss the issues around the island. They also go around door-to-door and spread the information about safety and new policies they try to implement.” (*Indonesian business owner*) (Action situation 1)	“The association of smallholder aquatic food producers that we have is a unified group. The first benefit we received was that we managed to get access to the ice-making machine of the Bureau of Fisheries and Aquatic Resources. This hasn’t happened in our town before, to get direct access to post-harvest facilities. That was out target.“ (*Association leader, Paombong*) (Action situation 2)
Power within	“[in reference to earthquake recovery] …there was a transition period for paying for food and drinks on the island. Many businesses gave food and drinks for free for a week or two, on an honour system […]. Many tried to pay afterwards, but wouldn’t accept. Many businesses just wanted to help, and realized they were going to lose money, but helped everyone.” (Local NGO employee) (Action situation 1)	“By interacting well with others, there will be something to learn. The best source of learning is experience. Through experience, we will be able to achieve a more advantageous market arrangement… We will gradually find ways to get our initiative in fish marketing to take off… It is scary if we think about what might come, but while we are here, we will slowly learn and work together. (*Fish farmer, Paombong*) (Action situation 3)

Rule and norm-making for environmental management involves the formal regulation of aquatic food production activities to ensure that coastal resources are conserved and used sustainably. In this action arena, local institutions are formulated and implemented primarily by local government units in collaboration with representatives from fishers’ and fish farmers’ groups. Rules in this action situation influence rules in the second and third action situations. For instance, scope rules targeted at the outcome of tracking the movement of aquaculture produce (in action situation 1), influence authority rules (i.e. whether or not a middleman can sell) in the marketing of aquatic food (in action situation 2). Boundary rules which determine which associations are formally registered and recognized influence position rules in community associations in action situation 3.

The second action situation pertains to marketing of food which is directly related to the challenge of economic inequities in market arrangements. It involves small scale aquatic food producers who produce food and sell to middlemen (locally called consignacions). Informal market arrangements in the form of market norms which are based on tacit agreements structure market exchange.

The third action situation relates to self-organization by aquatic food producers. It involves producers who organized themselves into associations either based on shared geographic location, similar livelihoods, or shared goals (e.g., finding better markets). This action situation is directly influenced by rule and norm-making (action situation 1) because state-sponsored rules provide an incentive (e.g., better access to livelihood assistance) and enabling conditions (e.g., support in formally registering an organization) for the establishment of community-level associations. Elected leaders from these associations typically serve as representatives in rule-making spaces at the municipal level as well as higher scales. Thus, concerns of actors in action situation 3 may feed into, and indirectly inform discussions in action situation 1.

#### Rule and norm-making for environmental management

Environmental degradation and declining biodiversity in the coast have been key challenges in the case study. The primary institutions for local governance of coastal and marine resources, particularly aquatic food production, are the Municipal Fisheries Ordinances. The city of Malolos approved its fisheries ordinance in the year 2000, Hagonoy in 2009, and Paombong most recently in 2021. Formulating and implementing the ordinances depend on commoning processes. Each municipality has a Municipal Fisheries and Aquatic Resources Management Council (MFARMC) consisting of various stakeholders which deliberates on local regulations and takes responsibility for implementation.

The ordinances provide *power over* to local government units to regulate various issues in aquatic food production such as the problem of encroachment of large-scale fishers on municipal waters. However, the implementation of the rule largely depends on commoning processes in the form of collaborative and voluntary arrangements between local government units and local aquatic food producers. The *power with* that is generated in these collaborations enables the monitoring of municipal waters through the labour of deputized local fisher volunteers and funding from local governments. Beyond stipulations of the law, shared goals and harmonious social relations between local government and food producers are vital to the process. This also explains why during periods of transition from one elected politician to another, monitoring activities in the area have been paused until both parties gain familiarity with each other and re-start the funding and monitoring.

The webs of relationships between different governance actors have created emergent arrangements that shape this action situation. The Central Luzon Bureau of Fisheries and Aquatic Resources (BFAR) maintains a field station in the case study area and assigns Fisheries and Livelihoods Development Technicians (FLDTs) to work in fishing and fish farming communities. FLDTs liaise with local government units and with fishers’ and fish farmers’ organizations primarily to assess the performance of BFAR’s livelihood assistance projects. But beyond this, the local presence of FLDTs also catalysed the creation of informal information-sharing networks. Communication between FLDTs and BFAR officials enabled local fishers and fish farmers to more rapidly share information on red tide, fish kills, and damage to livelihoods from flooding. The ways through which social connections have facilitated commoning through shared activities, active communication, discovered overlapping purposes, and mutual responses (e.g., state actors provide material support to aquatic food producers, producers support state projects through participation) underpin the dynamism of local governance. Here, *power with* lies latent in social connections and is activated under certain conditions (e.g., when a shared challenge arises). This *power with* that is rooted in relationships, gives actors the *power to* act on various issues. For instance, both BFAR and local government units were better able to implement capacity-building projects targeted at environmental protection (e.g., green water technology training course) because of existing ties with fish farmers. In turn, in their own communities, trained and organized fish farmers were able to advocate for a more environmentally friendly aquaculture practice by shifting from the use of toxic sodium cyanide to the prescribed tea seed powder during fish pond preparation.

Conversely, de-commoning also occurred and was chiefly influenced by the *power over* held by large-scale, intensive aquaculture producers. Unregulated disposal of polluted water from intensive fishponds was perceived by small scale producers to have caused extensive fish kills in backyard ponds and the disappearance of marine species. The shared experience of being severely impacted by water pollution prompted fishers and fish farmers to collectively demand local government intervention. This exhibited *power with*, in which an alliance between smallholder fishers and fish farmers enabled individuals to tap into a collective agency. Despite collective demand and the impacts of pollution on numerous smallholder livelihoods and ecosystems, polluting aquaculture practices remained unmonitored and unregulated. The government’s inability to respond and regulate aquaculture to protect the environment was perceived by smallholders as a result of the *power over* held by large producers. In this case, power asymmetries or power of large-scale producers over local government and small scale producers leads to de-commoning by preventing any meaningful action to address water pollution and privileging the interests of powerful and rich actors over the many, but less powerful ones.

#### Marketing of aquatic food

Locally, middlemen have *power over* market transactions (Manlosa et al. 2021). They set the price of fish, and can adjust fish auction mechanisms to serve various purposes. They hold a powerful position in the market arena because of the multiple functions they serve for other market actors. For instance, middlemen provide financial services in the form of loans to smallholder producers.

BFAR sought to address the disadvantaged positions of smallholder producers in markets by constructing a Community Fish Landing Center (CFLC). BFAR viewed this new fish landing site as a potential catalyst for smallholder producers to learn about market negotiations and gradually strengthen their market position. To take advantage of this opportunity, fishers and fish farmers from different community associations worked together to establish the Nagkakaisang Samahan ng mga Mangingisda ng Paombong/United Group of Fish Producers of Paombong (NASAMAPA) to be the state actors’ primary partner in operating the facility. Commoning here involves the shared interest in establishing a more advantageous market position. The organized status that the producers had already achieved led them to pursue further initiatives including applying for government grants to purchase storage and transport equipment and to seek out other markets away from their towns. Members of the organization established a cooperative and pooled resources to make their exploration of other markets possible. In this way, the *power with* that was made possible by shared struggles and the social cohesion from membership in the same organization fostered the *power to* explore the creation or discovery of new market opportunities.

#### Establishment of community associations by small scale aquatic food producers

Robust associations of small-scale fishers and fish farmers have played an important role in the commoning processes described above, but the coming together of aquatic food producers itself is constituted by commoning. The establishment of community associations has been a long-standing practice in the area. However, the recent establishment of new community associations emerged as a response to the government’s requirement for producers to be organized and registered to be able to actively participate in livelihood assistance programs.

While associations had an instrumental significance to BFAR because they helped facilitate the identification of needs for livelihood assistance and served as connecting nodes to reach other aquatic food producers, the associations expanded in significance. Social connections in these associations catalysed rapid information sharing, borrowing of materials for their livelihoods, support in personal difficulties, collective action during a crisis such as the initial pandemic lockdowns, and other key activities related to rule and norm-making and marketing as described above. Members of associations stayed connected through regular meetings which involved discussions on various issues but were just as importantly about being physically present, and sharing a meal together. These interactions within and outside of formal governance spaces fostered sharing in broader processes beyond aquatic food, and has sustained communal sharing, of being in relationship with one another, which constitutes commoning.

## Discussion

In our view, the commons field has evolved from a narrative of ‘tragedy of the commons’, to a narrative of ‘governing the commons’. This may be evolving into a third narrative, one focussed on ‘commoning the governance’. Power plays an important role in these processes, both analytically in understanding actor behavior and choices, and practically for understanding needed institutional changes to achieve desired outcomes. These social processes are commons themselves (i.e., second-order social commons; Partelow et al. 2021), as they need to be actively provisioned through collective action to enable governance activities to develop and mature. Thus, commoning puts forth community-based social processes as a central feature of governance, a form of co-production valued for its integrative processes. In our case analyses, we have seen that action arenas are where governance activities and thus commoning are situated, making the NAS approach useful in positioning commoning analytically.

Our review has attempted to show the similar but distinct perspectives on commoning, highlighting the general lack of power, while suggesting plural integration among sociological, institutional and human geography perspectives to build a dynamic analytical toolbox. For example, theories of bounded rationality and new institutional economics helped bridge the gap between rational choice views on human behavior with institutional theories suggesting rules and norms are more influential than cost–benefit choices with incomplete information (Levi 1990; Ostrom 1998). Critical institutionalism adds sociological understandings of institution construction, the role of power and the normative goals such as social justice and inequality (Cleaver and De Koning 2015; Clement et al. 2019; Nightingale 2019). We argue both are needed, and that commoning is a concept with the potential to aid their analytical integration. However, we argue it is clear that commoning activities cannot be disconnected from expressions of power, and thus integrating an explicit framework for power helps clarify how commoning adds further value to commons literature and where more focus is needed.

Nonetheless, synthetic approaches to studying society are not new, as seen in political economy research (Gibson et al. 2005) or philosophies such as critical realism (Gorski 2013; Longo et al. 2021). Where commoning fits, it can be seen as a close extension to critical realist philosophy in seeking analytical pluralism, trying to recognize the interpersonal experiences and social dynamics such as power as perhaps difficult to observe but important in shaping institutional development (Bennett et al. 2018), change and system outcomes. However, focussing on power in commons and commoning activities is, in our view, the least evolved or examined element of the commoning concept. This is not without some understandable reasons. Power dynamics can often only be felt or understood by an individual, not being easily observable from the outside. Power can have an influence on actor behavior beyond identifiable formal ontologies (i.e., the many frameworks that exist) which are the dominant analytical tools. Below we discuss the four expressions of power in our case study contexts, insofar as they advance the concept of commoning.

### Power over

Power over others, whether as an individual or a group, is perhaps the most widely recognized form of power. Commoning activities that arise in the context of power over can be seen when power over a group is controlled or utilized in a way that lacks abuse of that power, fosters open dialogue and inclusion over the less powerful to be recognized and valued in the relevant processes of governing despite the power difference. For example, on Gili Trawangan, most decision-making and resource allocation processes indicate power over locals, and many well established foreign business owners have economic power over employees and locals as well. Nonetheless, there is collective recognition that local Indonesian residents and employees are highly valued, need to be respected and included in much of the island’s decision-making, and actively supported when needed, such as in the August 2018 post-earthquake aftermath where many owners continued to pay salaries for months despite no business revenue (Partelow 2020). In Bulacan, the government’s power over local aquatic food producers provided incentive and enabling conditions for the self-organization of small scale aquatic food producers (Manlosa et al. 2021). This, in turn, precipitated beneficial community-level initiatives in aquatic food production and marketing. Power over is important for developing the concept of commoning, because identifying how and where it exists in action situations can help identify how the powerless can avoid abuses of power through being recognized, valued and included. Even if power over is maintained, it can be fruitful and constructive when done in a way that works towards minimizing the costs of collective action and empowering all involved (Chambers 2006). Conversely, attention to power over helps understand and determine mechanisms of de-commoning, when zero-sum power held by a few leads to exclusion, marginalization, or coercion.

### Power with

Power with is at the core of commoning. Being able to identify governing processes that distribute responsibilities and decision-making creates the opportunity for recognition of not only what is observable in terms of rules, harvesting, provisioning activities or outcomes, but also what role the social processes rooted in the experienced realities of those involved play in guiding behavior in action situations. The formation of coalitions or associations can also be used to aggregate power into larger coordinated groups, which may undermine ambitions of pursuing commoning strategies of co-created processes if those groups become further polarized due to the aggregation and sunk costs, making them inflexible and exclusive. On Gili Trawangan, SCUBA dive business aggregated their power to form multiple associations as an effort to share power and decision-making responsibilities. These community-based organizations have helped shape the rules and outcomes in their respective action situations, arguably in a positive direction. Nonetheless, some actors have avoided such efforts, and actively capitalized on collective gains without contributing back. Furthermore, aggregate power with, on Gili Trawangan, has created resistance among those who have felt less included and have skepticism that some individuals with power are self-interested. In Bulacan, the formation of associations by small scale producers provided the basis for collective action including advocacies for environment-friendly aquaculture practices, explorations for more equitable market-arrangements, and mutual support for one another’s livelihoods. It also provided smallholders with a legitimacy that enabled easier access to government support and services. In sum, the case studies demonstrate that the mechanisms through which power with is exercised and the extent to which it promotes inclusion shape whether social processes can be characterized as commoning or de-commoning processes.

### Power to

Power to, according to VeneKlasen and Miller (2007) refers to the “unique potential of every person to shape his or her life and world […], opens up the possibilities of joint action […and that] citizen education and leadership development for advocacy are based on the belief that each individual has the power to make a difference”. Power to change and take action is essential for commoning, and rooted in perceived abilities and prior experiences of individuals despite the influence on them from surrounding institutions and economic costs and benefits. On Gili Trawangan, coral reef use and waste management issues have been approached through collective action of individuals involved over many years and have been catalysed by developing education and empowerment opportunities for businesses to be more sustainable and to participate in the island’s future. Power also needs to be recognized and valued by other action situation participants in many cases to be effective. In Bulacan, commoning processes enabled smallholder producers to tap into a collective power to, which otherwise would have been absent. For instance, commoning from smallholder associations not only facilitated discussions on shared livelihood challenges such as being disadvantaged in market arrangements, but also fostered resource-sharing and collective agency to address challenges, for instance through collectively exploring more favourable markets. Hence, commoning plays a vital role in increasing collective power to, which is important for social groups to achieve shared goals.

### Power within

Belief in one’s self is an essential feature for being able to pursue your needs, those of your family and community. This includes the ability to recognize individual differences while respecting others (VeneKlasen and Miller 2007). VeneKlasen and Miller (2007) further state that power within refers in part to perceived agency, and “is the capacity to imagine and have hope; it affirms the common human search for dignity and fulfilment, [and that] many grassroots efforts use individual storytelling and reflection to help people affirm personal worth,” (p. 45). On Gili Trawangan, the most important shared power within is the confidence in the social infrastructure developed over years of self-organization. The years of experience growing the island’s economy and dealing with disasters and hardships have shaped individual agency and perceived opportunity and resilience of many longer residents. This collective confidence is being perceived as being eroded as the island grows and attracts more outside investors without the history of collective action. While in Bulacan, power within was reflected in smallholder producers’ sense of being legitimately recognized as a government partner, a sense of having a voice and of being heard, a recognition of the individual’s ability to be a valuable part of a larger group and to contribute. Each of the efforts by smallholder producers discussed in earlier sub-sections were preceded by a belief that the challenges people encountered in their livelihoods can be acted on and potentially changed. While commoning plays out at a collective level and power within at the individual level, both are connected and can be mutually reinforcing as the case studies show. Commoning can foster people’s power within by enabling people to be part of broader and shared social processes to which they can contribute and accomplish more than they would be able to do alone. At the same time, people’s power within may also be strengthened as commoning provides opportunities for individuals to exercise and strengthen their power within.

### Advancing the networks of action situation perspective with commoning and power

The network of action situations approach continues to be of value in understanding polycentric system configurations and actor interactions (Carlisle and Gruby 2019; Kimmich et al. 2022), or more broadly, how sets of institutions interact and co-shape each other in environmental governance (Partelow et al. 2020). However, while detailed and descriptive in decomposing the constituent parts of governance, it can miss the spaces in between where experienced reality exists and drives behavior combining rules, norms and rationalism and while recognizing influences beyond these factors. Here we argue that commoning processes can be useful in foregrounding those often unseen and un-analysed dynamics in commons literature and act as a boundary object (Clement et al. 2019) to integrate normative concepts such as power and justice into institutional analysis through a pluralistic and interdisciplinary perspective. This can also link to the individual level and provide a frame for analysing if individuals perceive processes to be shared, co-produced or co-created, in other words, developed through commoning. We have attempted to demonstrate this value in the case studies above, however, we recognize that these analyses are limited to the basic analytical components of the network of action situations approach, and does not fully explore all aspects of the network approach or provide a nuanced analysis of actor positions and rules in our link to commoning. However, we encourage more detailed analyses.

## Conclusions

In closing, commoning can embody a wide range of activities and processes, however, because the concept is still at an early stage of development and application, it can pose challenges in definitions and applications, and other concepts will likely be useful to help unpack the role commoning plays in other case studies. This article provided one perspective on the concept, and added a new dimension through reviewing the literature, analysing two case studies, and focussing on power. We believe that this article helps demonstrate that commoning can be a useful bridging tool or boundary object for integrating different parts of commons scholarship and institutional analysis, particularly finding new ways to include aspects of sociology and critical institutionalism into new institutional economics and political science approaches. Boundary concepts are useful because they find common ground, often using new terminologies and framings to avoid disciplinary discourses that are often rooted and historically associated with different fields or disciplines of scholarship.
